# Microbial carbohydrate-active enzymes influence soil carbon by regulating the of plant- and fungal-derived biomass decomposition in plateau peat wetlands under differing water conditions

**DOI:** 10.3389/fmicb.2023.1266016

**Published:** 2023-09-04

**Authors:** Mingyao Xiong, Wei Jiang, Shuzhen Zou, Di Kang, Xianchun Yan

**Affiliations:** Key Laboratory of Southwest China Wildlife Resources Conservation (Ministry of Education), China West Normal University, Nanchong, China

**Keywords:** eastern margin of the Tibetan Plateau, soil enzymes, CAZyme, plant-derived carbon, metagenomics

## Abstract

Peatlands are important carbon sinks and water sources in terrestrial ecosystems. It is important to explore their microbial-driven water-carbon synergistic mechanisms to understand the driving mechanisms of carbon processes in peatlands. Based on macrogenomic sequencing techniques, located on the peatland of the eastern margin of the Tibetan Plateau with similar stand and different water conditions, we taken soil properties, microbiome abundance, CAZyme abundance and enzyme gene pathways as the object of study, investigated the characterization of soil microbial carbohydrate-active enzymes (CAZymes) under different water gradients in peatland. According to the results, these three phyla (Chloroflexi, Gemmatimonadetes, and Verrucomicrobia) differed significantly between water gradients. Under dried wetlands, the abundance of CAZymes involved in hemicellulose and glucan degradation increased by 3.0 × 10^−5^ and 3.0 × 10^−6^, respectively. In contrast, the abundance of CAZymes involved in chitin degradation decreased by 1.1 × 10^−5^ (*p* < 0.05). It highlights that regulating plant- and fungus-derived carbon metabolism processes by soil microorganisms in highland peatlands is a crucial mechanism for their response to water changes. Most plant-derived carbon fractions are regulated by soil enzymes (endo-beta 1,4-xylanase, alpha-L-arabinofuranosidase, and alpha-L-fucosidase) containing CAZymes functional genes. Additional findings in this enzyme gene pathway indicate that water changes that affect soil carbon fractions indirectly influence the three enzyme gene metabolic pathways related to plant carbon sources (the glycolysis/gluconeogenesis, other glycan degradation and amino sugar, and nucleotide sugar metabolism). Overall, this study highlights the significance of microbial CAZymes in highland peatland soil carbon processes and indicates that microbial conversion of plant and fungal biomass carbon is more sensitive to water changes.

## 1. Introduction

According to the global peatland assessment report published in 2022, the global peatland area is approximately 4.88 × 10^6^ km^2^ (UNEP, 2022), and the accumulated carbon accounts for approximately one-third of the total global soil carbon pool (Scharlemann et al., 2014). Peatlands are essential carbon sources and sinks for terrestrial ecosystems due to their ability to store and fix carbon for extended periods without disturbance (Moore and Knowles, 1989; Limpens et al., 2008). The formation of peatlands is slow and dependent on anaerobic and acidic conditions in wetland environments. However, global warming is redistributing global precipitation, and the massive melting of snow and ice at high altitudes may reduce river runoff, causing to water scarcity and degrading of wetland environments in highland wetlands and posing unknown risks to peatlands (Schoning et al., 2005). In peatlands, a large amount of plant residues are deposited to form a peat layer that reduces the intensity of organic matter degradation (Joosten and Clarke, 2002), promotes the accumulation of carbon and slows down its geologic circulation, which helps to slow global warming (Joseph, 2005; Wang, 2009). Microbial carbohydrate-active enzymes (CAZyme) play a significant role in the degradation of soil organic matter, regulating soil organic carbon content via changes in soil organic matter content and, consequently, regulating atmospheric carbon dioxide concentrations (Cantarel et al., 2009; Zhao et al., 2020). Several studies have been conducted in different regions and environments to determine the importance of water conditions for carbon cycle functioning in peatlands, but varying conclusions and opinions remain (Tamkevičiūtė et al., 2018; Tipping et al., 2021). The relationship between water changes and CAZyme is poorly characterized, and the coordination of different water conditions on carbon fractions and CAZyme in high-cold peatlands has yet to be investigated comprehensively and systematically.

Prior research has primarily focused on the effects of environmental factors such as pH and climate on peatland carbon function, in the context of global warming. For example, previous studies found that precipitation frequency and precipitation variability indirectly regulate peatland carbon emissions by affecting dissolved organic carbon (DOC) content (Xiaoya et al., 2018; Dong et al., 2021). In addition, environmental factors such as pH, temperature, and vegetation communities have been shown to have a substantial impact effects on peatland carbon emissions (Bergman et al., 1998; Dinsmore et al., 2009; Helbig et al., 2019; Zhang et al., 2019). In recent years, in addition to environmental factors, CAZymes have been demonstrated to help investigate the functional response of microorganisms to the carbon cycle, and specific glycoside hydrolases (GHs) and auxiliary activities (AAs) have been associated with the degradation of biomass, such as polysaccharides and lignin (Lladó et al., 2019; Lopez-Mondejar et al., 2020). For example, cellulases or hemicellulases in the family of GHs primarily degrade plant biomass, whereas lysozyme and chitinase are involved in the degradation of dead biomass in bacterial and fungal communities, respectively (Vincent et al., 2014). However, the role of carbohydrate-activated enzymes (CAZyme) in the function of carbon cycling in high-altitude peatlands has not been reported, and the gene abundance of CAZyme interacts with the degree of degradation and soil depth, and the gene abundance of CAZyme is influenced by soil degradation and depth. It is also possible that they do not share the same water conditions, which then affect the dynamic changes of the enzyme to indirectly affect the carbon cycle (Žifčáková et al., 2017; Zhang Q. et al., 2023). Different degrees of succession in peatlands caused by differences in soil water changes led to changes in enzyme activity and nutrient content (Jing et al., 2022; Yu-ting et al., 2022) and plant litter, root secretions, and soil humus decomposition are all impacted by differences in soil water (Chen et al., 2018; Zhang et al., 2020).

In this study, soil samples were gathered from peatlands on the eastern margin of the Tibetan Plateau that had similar geographical site conditions but distinct water regimes. We studied the dynamic changes in microbial carbohydrate-active enzymes (CAZyme) associated with soil carbon cycling in peatlands under various water conditions. We aim to provide a theoretical foundation for elucidating the mechanism of the influence of water changes on soil carbon processes in peatlands against the backdrop of global change, as well as data support for the conservation and restoration of peat wetlands at the eastern edge of the Tibetan Plateau. Based on previous research findings, we propose the following hypotheses: (1) a reduction in water can influence the composition of soil carbon fractions in peatlands; and (2) water conditions regulate the degradation process of soil organic carbon through the abundance and function of carbohydrase. This study contributes to a greater comprehension of the response mechanisms of peatland carbon dynamics to environmental changes along the eastern margin of the Tibetan Plateau. This study also contributes to the future elucidation of the microecological underlying the mechanisms responses of peatland to global change.

## 2. Materials and methods

### 2.1. Study area

**Figure 1:**
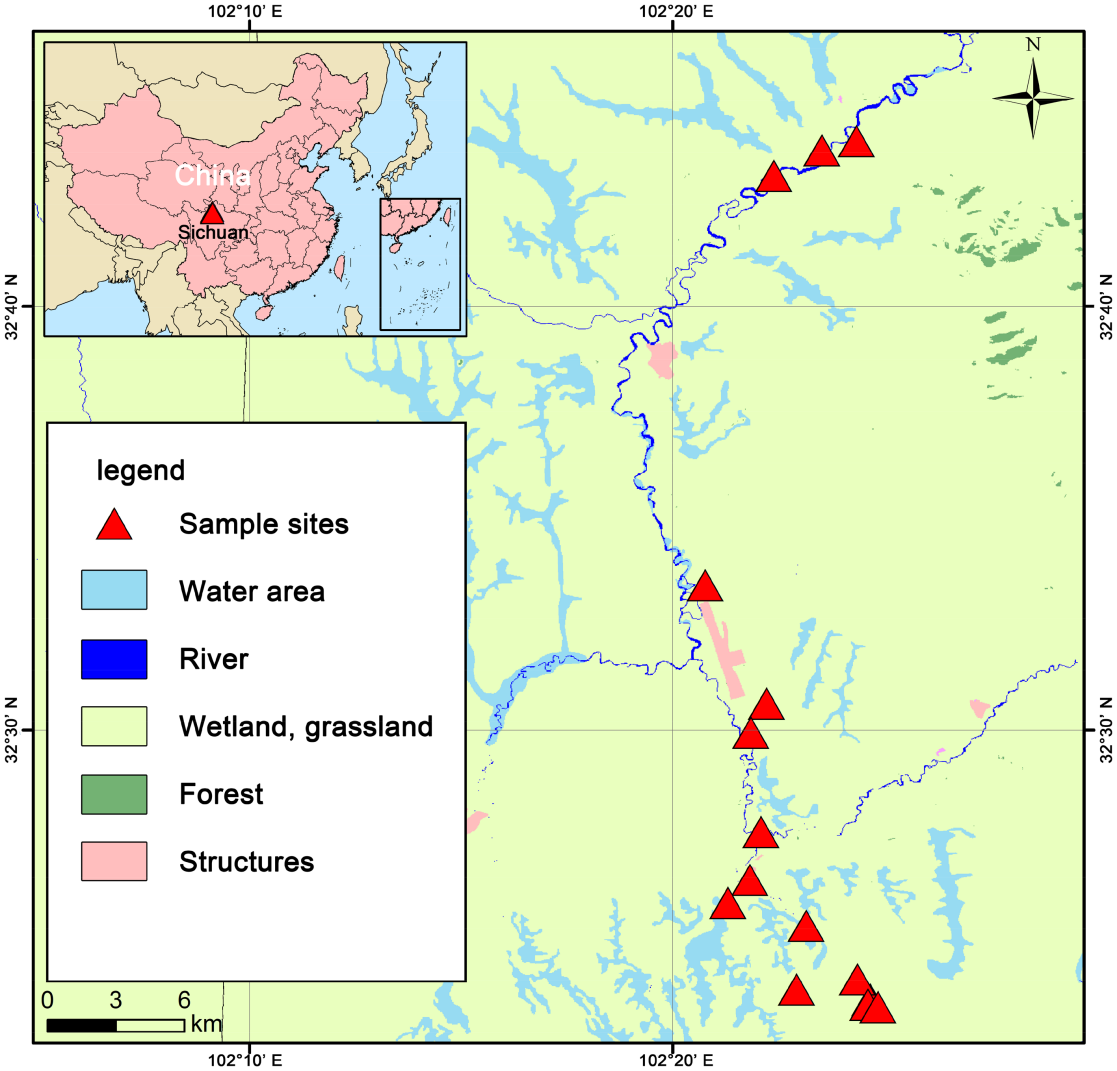
Distribution of sample sites in the eastern Tibetan Plateau region.

The study site is located in the eastern fringe of the Tibetan Plateau (32.73° ~ 32.39° N, 102.34° ~ 102.41° E) (Figure 1), with an average elevation of 3,400–3,800 m and a total area of approximately 36,970 km^2^. The study area has a plateau subduction zone semi-humid continental monsoon climate. The mean annual temperature (MAT) is approximately 0.9 ~ 2.5°C, and the mean annual precipitation (MAP) is approximately 518 ~ 800 mm. There is a growing season and a non-growing season, with the growing season being short (July–September), and the cold non-growing season basically occurs year- round. The peat wetlands at the eastern edge of the Tibetan Plateau is a typical plateau peatland in Asia, rich in peat storage, and the largest plateau swamp concentration area in China (Xin-Tong et al., 2019). The peat wetlands at the eastern edge of the Tibetan Plateau have a complete ecosystem structure, with well developed vegetation, and the peatland vegetation is mainly dominated by swamp vegetation and alpine meadows.

### 2.2. Experimental design and soil sampling

After conducting an investigation, 15 sample sites were selected, representing three different water conditions: water-rich wetland (F), water-scarce wetland (IF), and dried wetland (D). In order to make the sites similar, the selected sites were all sedge grasslands (dominant species were *Carex hongyuanensis*, *Carex muliensis* and *Carex setschwanensis*, and the main associated species were *Elymus nutans*, *Artemisia capillaris*, *Melilotoides ruthenicus* and *Artemisia capillaris*). At each of the sample sites, 10 sampling points with random locations were selected for soil sampling. Visible plants and litter were removed before soil collection, and then the soil at a depth of 0 ~ 10 cm was thoroughly mixed before sampling. Deep water should be avoided areas when sampling, and if shallow water areas are encountered, samples should be taken in the same way after draining water. Subsequently, 10 samples were thoroughly mixed to make composite soil samples, and fine roots and large plant debris were removed from the composite soil samples. One part of each sample was used to analyze the basic properties of the soil, the other part was used to determine soil respiration and temperature sensitivity (50 g fresh weight), and the remaining part was stored at −40 degrees Celsius for subsequent metagenomic sequencing.

### 2.3. Measurement of soil carbon fractions and their chemical properties

We determined dissolved organic carbon (DOC), total soil nitrogen (TN), dissolved organic nitrogen (DON), soil organic carbon (SOC), C/N. Dissolved organic carbon (DOC) was extracted by MK_2_SO_4_ solution and determined by a TOC-TN analyzer (Shimadzu, Kyoto, Japan) (Shi et al., 2018). Total soil nitrogen (TN) was analyzed and determined by the Kjeldahl method using concentrated sulfuric acid and mixed catalyst decoction. The dissolved organic nitrogen (DON) content was obtained by the difference between soil soluble total nitrogen and soil inorganic nitrogen, and soil soluble total nitrogen, ammonium nitrogen and nitrate nitrogen were also determined by the Kjeldahl method. Soil total carbon (TC) and soil organic carbon (SOC) were determined by the external heating method using H_2_ SO_4_-K_2_CrO_27_ (Shidan, 2000). C/N is the ratio of soil organic carbon (SOC) to total soil nitrogen (TN). Soil cumulative respiration (*C_um_*) is first phenolphthalein as an indicator, HCl titration to neutralize the remaining NaOH, so that the generation of Na_2_ CO_3_ transformed into NaHCO_3_, and then methyl orange as an indicator, HCl titration, so that all the NaHCO_3_ are changed into NaCl, methyl orange as an indicator when the amount of HCl consumed by two times, that is, the neutralization of the Na_2_ CO_3_ dosage, so that the absorption of the CO_2_ amount can be calculated. And finally the *C_um_* value by the soil respiration formula. Specific experimental methods for measurement of *C_um_* are described in Supporting Material Part S1. The soil respiration calculation formula is as follows:


(1) $$C_{um}=\frac{\sum_{i=1}^{n}\left(C_{t}-C_{b}\right)\times V\times 10^{-3}}{W}$$

Where *C_um_* is the cumulative soil respiration (mgC-CO_2_/g); C_t_ is the carbon content in lye from different treatments (mgC-CO_2_/L); C_b_ is the carbon content in blank lye (mgC-CO_2_/L); V is the volume of lye (mL); and W is the soil weight (g).

### 2.4. DNA extraction, gene prediction and non-redundant gene set construction

Total community genomic DNA extraction was performed from 15 soil samples using the E.Z.N.A. Soil DNA kit (Omega, M5635-02, United States) following the manufacturer’s instructions. We measured the DNA concentration using Qubit 4.0 (Thermo, USA) to ensure that sufficient amounts of high quality genomic DNA were extracted.

A total of 500 ng of DNA from each sample was used as input material for DNA sample preparation. Sequencing libraries were generated using the Hieff NGS^®^MaxUp II Erie^®^ DNA Library Preparation Kit (12200ES96, YEASEN, China) according to the manufacturer’s instructions and index codes were added to the attribute sequences of each sample. Pairwise sequencing of this library was performed on a NovaSeq 6000 sequencer (Illumina, USA) (Modi et al., 2021).

The ORFs of the splicing results were predicted using pre-cut proteins (version 2.60), and genes with lengths greater than or equal to 100 bp were selected and translated into amino acid sequences. For the gene prediction results of each sample, a non-redundant gene set was obtained using CD-HIT (version 2.60). Salmon (version 1.5.0) was used to construct a specific index for the non-redundant gene set, a biphasic algorithm and a constructed bias model were used to accurately quantify the gene abundance in each sample, and gene abundance was calculated based on the gene length information.

### 2.5. Macrogenome assembly

First, multi-sample hybrid splicing was performed using Megahit (version 1.2.9) to obtain initially spliced contiguous sequences. Reads were then cleaned up using bowite2 (version 2.1.0), mapped back to the splicing results, unmapped reads extracted, and spliced again using SPAdes (version 3.13) to obtain low abundance sequences. A series of bins were loaded using MetaWRAP (version 1.3.2) and the processes of garbage can classification, garbage can purification, garbage can quantification, garbage can reorganization, and garbage can identification were performed sequentially. After filtering, a genome sketch of a single bacterium with high integrity and low.

### 2.6. The annotation of CAZyme

CAZy (Carbohydrate-Active Enzymes Database) is a specialized database of carbohydrate-active enzymes, including related enzyme families that catalyze carbohydrate degradation, modification, and biosynthesis. This paper focuses on glycoside hydrolases (GHs) and auxiliary activities (AAs). The gene set protein sequences were aligned with the CAZy database using HMMER3 to obtain the annotation information of their corresponding carbohydrate-active enzymes. The screening conditions were E-value<1e-5 and Score > 60. and the abundance of each functional level of CAZy was counted.

### 2.7. Statistical analysis

One-way analysis of variance (ANOVA) was used to assess the effects of changing water conditions on soil carbon fractions, soil physicochemical properties, CAZyme families, carbon degrading enzyme-associated microbial phyla and CAZyme functional genes. All tests were performed using SPSS 22 (IBM, Armonk, USA). Pearson’s analysis was used to screen for enzymes containing CAZyme functional genes that were primarily and significantly associated with soil carbon fractions and to further analyze the relationship between degrading enzymes and soil physicochemical properties. *P* < 0.05 was considered as statistically significant difference. Redundancy analysis (RDA, Canoco 5.0, Microcomputer Power, New York, NY, USA) was used to investigate the relationship between specific environmental factors and degradative enzyme gene pathways based on the major enzyme gene pathways of degradative enzymes affecting soil carbon fractions. Pearson analysis heatmaps and RDA plots were drawn in Origin 2021.

## 3. Results

### 3.1. Effect of water conditions on biochemical properties of soils in upland peat wetlands

**Figure 2:**
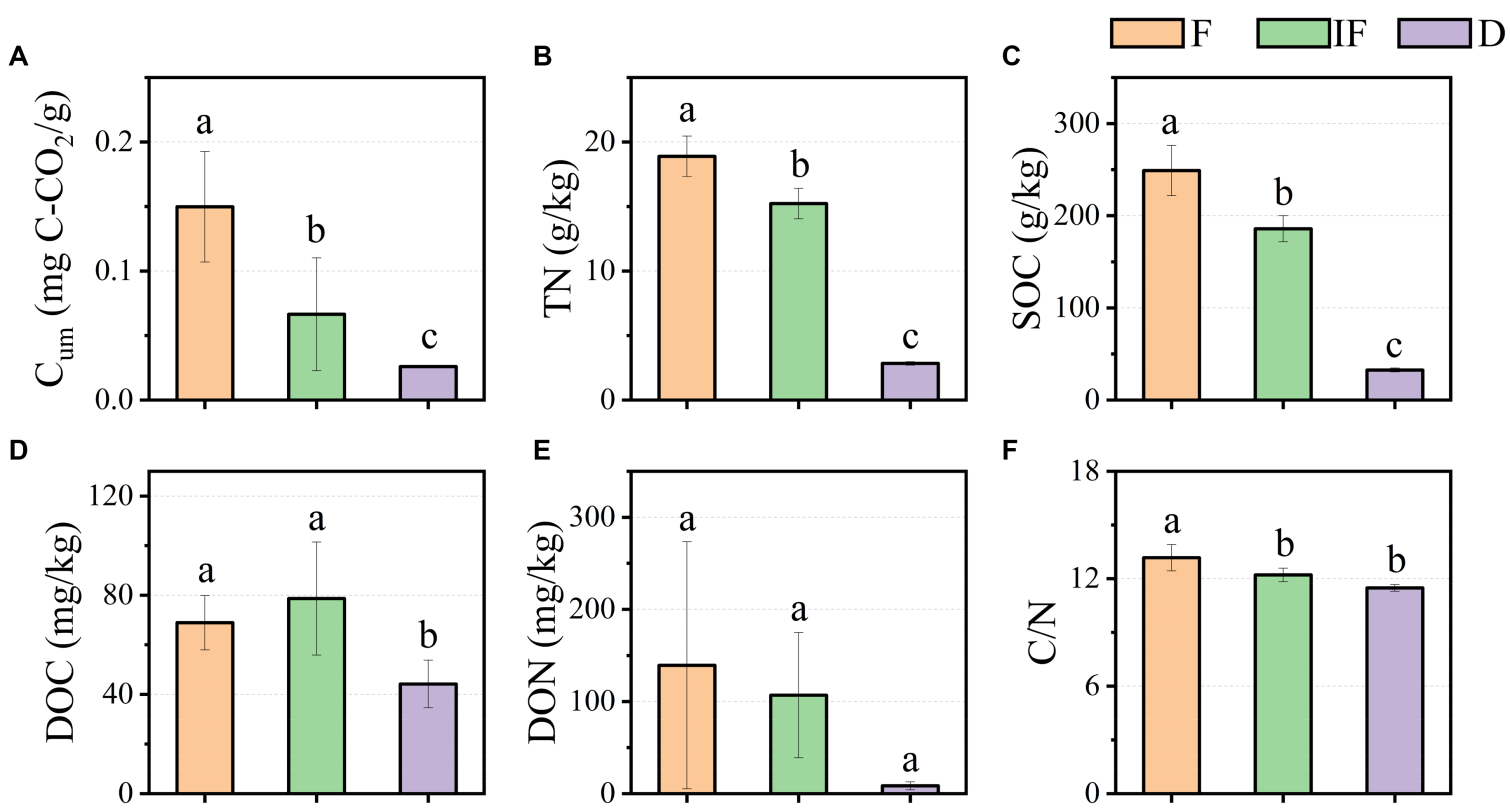
Effect of water conditions on soil biochemical properties of upland peatland. **(A)** Cum. **(B)** TN. **(C)** SOC. **(D)** DOC. **(E)** DON. **(F)** C/N. F is Water-rich wetland. IF is Water-scarce wetlands. D is Dried wetland. *C_um_* is cumulative soil respiration; TN is total nitrogen; SOC is soil organic carbon, DOC is dissolved organic carbon; DON is dissolved organic nitrogen; C/N is the ratio of SOC to TN.

As shown in Figure 2A, *Cum* ranged from 0.026 mg C-CO_2_/g-0.15 mg C-CO_2_/g in the water-rich wetland, water-scarce wetland, and dried wetland; meanwhile, soil TN was 2.837 g/kg-18.894 g/kg, and SOC was 32.586 g/kg-249.003 g/kg, respectively (Figures 2B,C). Soil DOC varied from 44.188 mg/kg-78.634 mg/kg (Figure 2D). Similarly, DON varied from 8.577 mg/kg-139.305 mg/kg and C/N was 11.480–13.171 (Figures 2E,F). *C_um_*, TN and SOC varied significantly with water under different water conditions and increased significantly with decreasing water content (Figures 2A–C, *p* < 0.05). DOC increased under water-scarce wetlands but DOC decreased under dried wetland conditions and varied significantly with water-rich wetland and water-scarce wetland conditions (Figure 2D, *p* < 0.05). C/N varied significantly with C/N decreasing with decreasing water content, which was positively correlated with changes in water content and differed significantly with water-scarce wetland and dried wetland conditions (Figure 2F, *p* < 0.05). DON decreased with decreasing water content, but changes in water conditions did not significantly affect DON (Figure 2E, *p* < 0.05). In this study, only DOC showed a tendency to increase in content under water-scarce wetland treatment conditions by 9.717 mg/kg (*p* < 0.05), while the values of other soil biochemical indicators were highest under dried wetland conditions and gradually decreased with decreasing water content (Figure 2).

### 3.2. Changes in SOM degradation-related CAZyme families and functional genes under different water conditions

**Figure 3:**
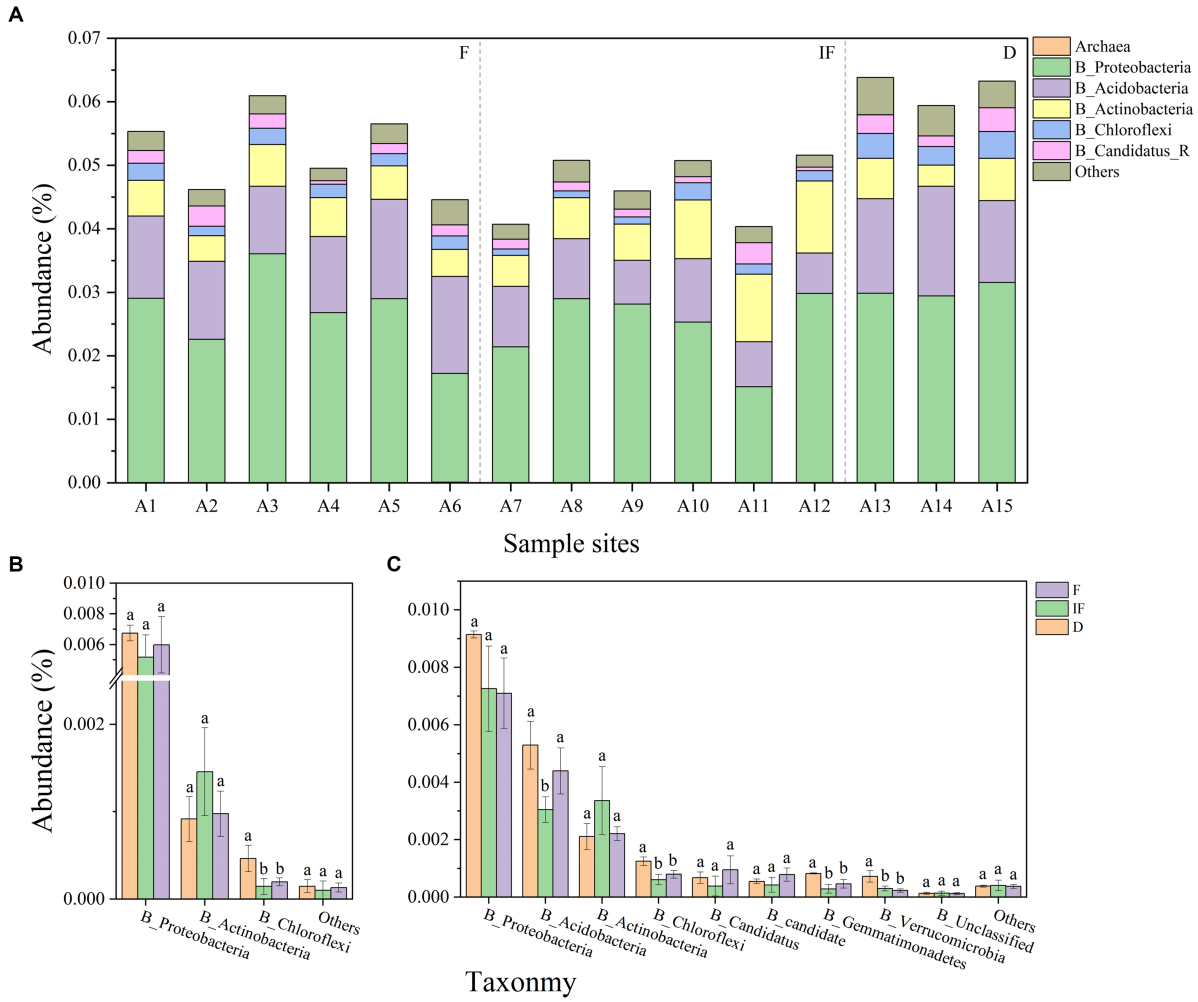
**(A)** Abundance of microbial phyla associated with carbon-decomposing enzymes for each sample. **(B)**-AA phylum differences, **(C)**-GH phylum differences; abundance less than 0.01 percent for others.

From the 15 soil samples collected, it was found that the microbial phyla associated with carbon-decomposing enzymes were predominantly attributed to the bacterial community, with only less than 0.01% being other microbial phyla. Among them, Proteobacteria, Acidobacteria and Actinobacteria were among the dominant phyla in the bacterial community, while Archaea had the smallest relative abundance in the bacterial community (Figure 3A). In addition, most of the GH and AA families in all CAZyme pools were mainly explained by bacterial communities, and targeting carbon catabolism-associated GH, and AA microbial phyla produced significant differential changes under different water conditions. Among the AA microbial phyla Proteobacteria and Chloroflexi both showed the highest microbial phyla abundance under dried wetland conditions, with the highest abundance of Proteobacteria and Chloroflexi being 0.0068 and 0.0009%, respectively, while Actinobacteria showed the highest microbial phyla abundance under water-scarce wetland conditions, with the highest abundance of Proteobacteria and Chloroflexi being 0.0009%. phylum showed the highest abundance with the highest abundance of 0.0015%, but only Chloroflexi differed significantly between water-scarce wetlands and water-rich wetland conditions (Figure 3B, *p* < 0.05). Among the GH microbial phyla Proteobacteria had the highest abundance and showed a decreasing trend in microbial phyla abundance with gradual water increase, with a range of 0.009–0.007%, which was negatively correlated with changes in water content; Verrucomicrobia had the lowest abundance, which decreased in abundance with increaseing water, which was negatively correlated with changes in water conditions; Acidobacteria, Chloroflexi, Gemmatimonadetes, and Verrucomicrobia all decreased in abundance with increasing water compared to dried wetland conditions, and the differences were significant under water-scarce wetlands conditions (Figure 3C, *p* < 0.05). The highest abundance of the dominant phyla Proteobacteria and Acidobacteria was found in the soil under dried wetland conditions, and the highest abundance of the dominant phylum Actinobacteria was found under water-scarce wetland conditions.

**Figure 4:**
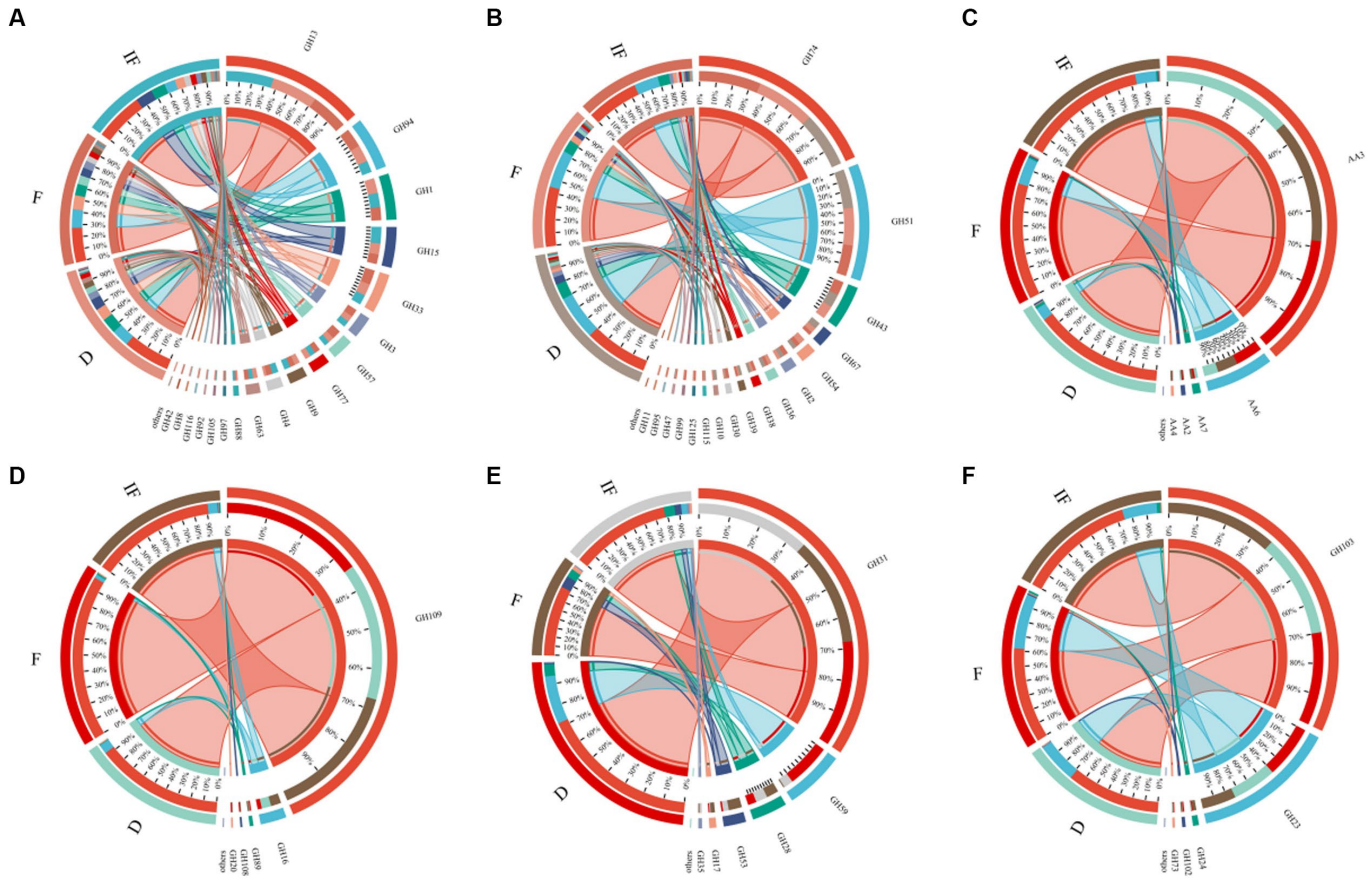
Relative abundance of functional genes based on the decomposed SOM type CAZymes. **(A)** Cellulose; **(B)** hemicellulose; **(C)** lignin; **(D)** chitin; **(E)** glucan; **(F)** peptidoglycan.

We selected specific CAZyme families involved in SOM catabolism, including plant-derived biomasses such as cellulose, hemicellulose and lignin; fungal-derived biomasses such as chitin and glucan; and bacterial-derived biomasses such as peptidoglycan. The functional classification of glycoside hydrolases (GH) and auxiliary activity (AA) involved in the degradation of plant-derived and microbial-derived components in all samples of this study is shown in Supplementary Table S1, with the GHs family involved in the breakdown of most of the different SOMs and the AAs family contributing mainly to the breakdown of the plant-derived biomass lignin (Figure 4). In plant-derived biomass degradation, the largest number of GHs family types were included in the degradation of cellulose and hemicellulose carbon sources, with the highest relative abundance of CAZyme functional genes being GH13 and GH74, respectively (Figures 4A,B); in the degradation of lignin carbon sources involved in the degradation of lignin carbon sources, the functional genes of CAZymes were dominated by AA3 (Figure 4C). In biomass decomposition such as fungal-derived chitin and glucan, clusters GH109 and GH31 contributed the most, respectively (Figures 4D,E). GH103 and GH23 mainly controlled bacterial-derived biomass degradation (Figure 4F).

**Figure 5:**
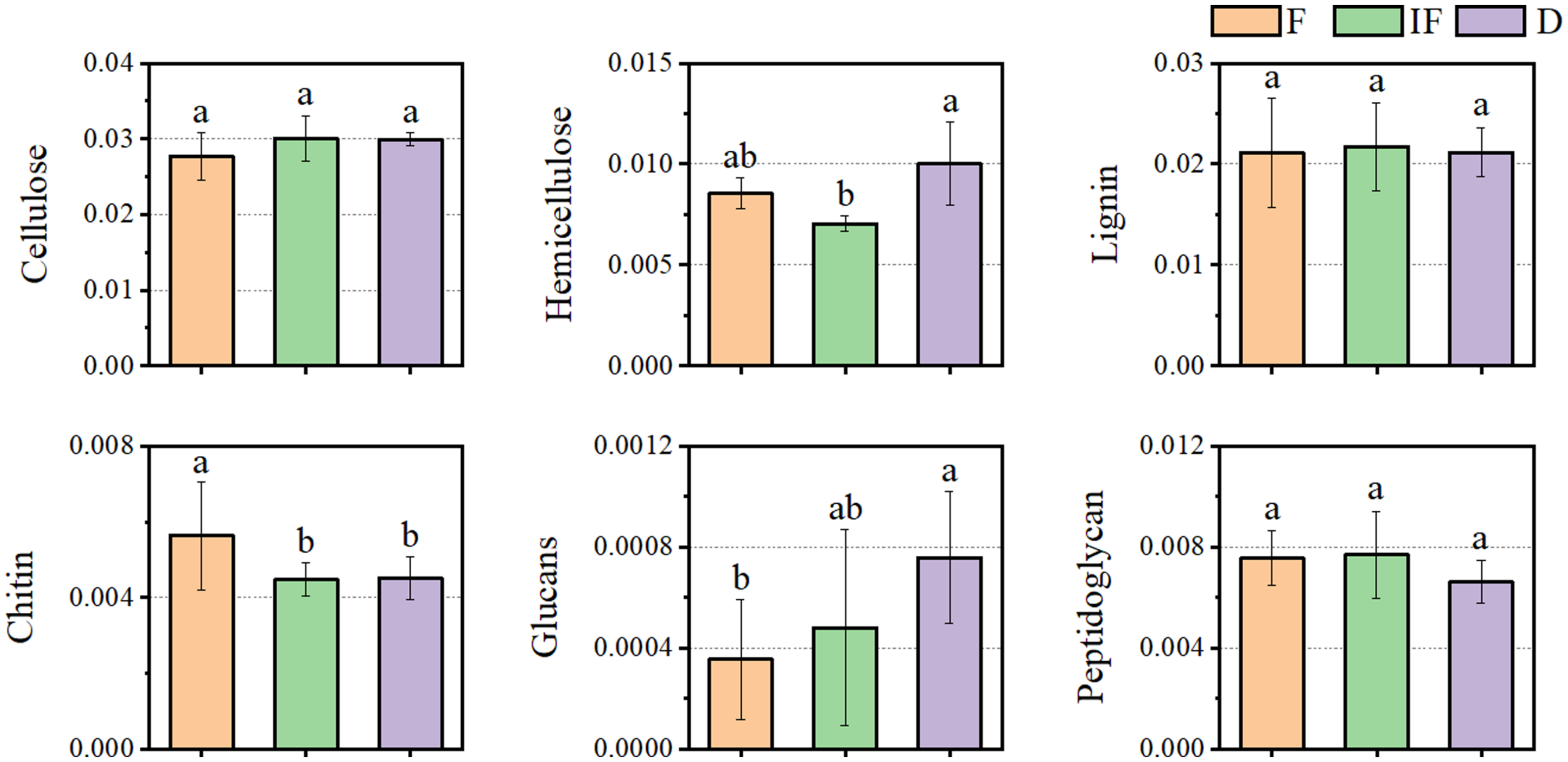
Differences in the functional gene abundance of CAZymes based on decomposed SOM types.

As shown in Figure 5, most of the functional genes of CAZymes involved in carbon degradation had the same trend for different SOM decompositions. They mostly increased under dried wetland conditions compared to water-rich wetland conditions. In addition to the main direction of change, there were also a few types of carbon catabolism with different variation differences. For the case of chitin and peptidoglycan degradation, the abundance of CAZyme functional genes decreased under dried wetland conditions, and the difference in chitin changes was significant (*p* < 0.05). The relative abundance of CAZyme functional genes involved in hemicellulose degradation was significantly increased by 0.003% under dried wetland conditions compared with water-scarce wetland conditions (*p* < 0.05); in the case of glucan degradation, the abundance of CAZymes functional genes increased with the gradual decrease in water, which was negatively correlated with the change in water conditions, and the difference in the change in the relative abundance was significant in the dried wetland conditions (P & & lt; 0.05). Therefore, there was a significant effect of changes in water conditions on the abundance of CAZyme functional genes involved in the degradation of plant-derived biomass and fungal-derived biomass carbon sources.

**Figure 6:**
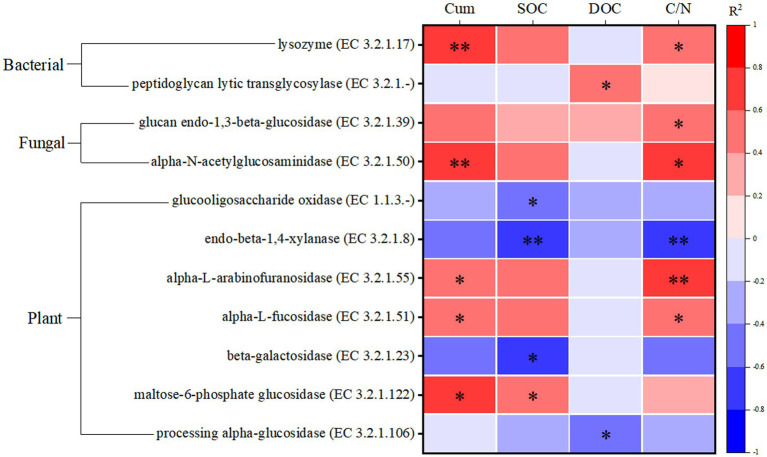
Screening of enzymes significantly associated with soil carbon fractions. *C_um_* is cumulative soil respiration; SOC is soil organic carbon; DOC is dissolved organic carbon; C/N is the ratio of SOC to TN. *p < 0.05, **p < 0.01.

The correlation of all CAZyme functional genes involved in different SOM degradation with soil carbon fractions in all samples of this study is shown in Supplementary Figure S1. As shown in Figure 6, enzymes containing CAZyme functional genes mainly involved in the role of degradation of soil carbon fractions were screened from them. Most of the enzymes with CAZyme functional genes involved in biomass degradation of plant origin showed significant negative correlation (*p* < 0.05) with SOC, whereas the enzymes with CAZyme functional genes involved in biomass degradation of fungal and bacterial origin showed significant positive correlation (*p* < 0.05) with cumulative soil respiration (*C_um_*) and C/N. Thus the relevant enzymes involved in the degradation of plant-derived biomass are significantly associated with the carbon fraction of highland peatlands. In addition, these soil enzyme carbon metabolic pathways mainly involved in carbon degradation are shown in Supplementary Table S2. maltose-6-phosphate glucosidase (EC 3.2.1.122) associated with the degradation of plant carbon sources and glucan endo-1,3-beta-glucosidase associated with the degradation of fungal carbon sources (EC 3.2. 1.39) which are both involved in the metabolism of starch and sucrose; peptidoglycan lytic transglycosylase (EC 3.2.1.-) associated with the degradation of bacterial carbon sources and alpha-N-acetylglucosaminidase (EC 3.2.1.50) associated with the degradation of fungal carbon sources are both inside the glycosaminoglycan degradation metabolic pathway; alpha-L-arabinofuranosidase (EC 3.2.1.55) that associated with the degradation of plant carbon sources and lysozyme (EC 3.2.1.17) that associated with the degradation of bacterial carbon sources, which are both in the amino sugar and nucleotide sugar metabolism (ASN) metabolic pathway, suggesting that these enzymes play an important role in participating in the metabolism of soil carbon fractions.

### 3.3. Influence of major enzyme genes for microbial SOM degradation on soil carbon fractions under changing water conditions

**Figure 7:**
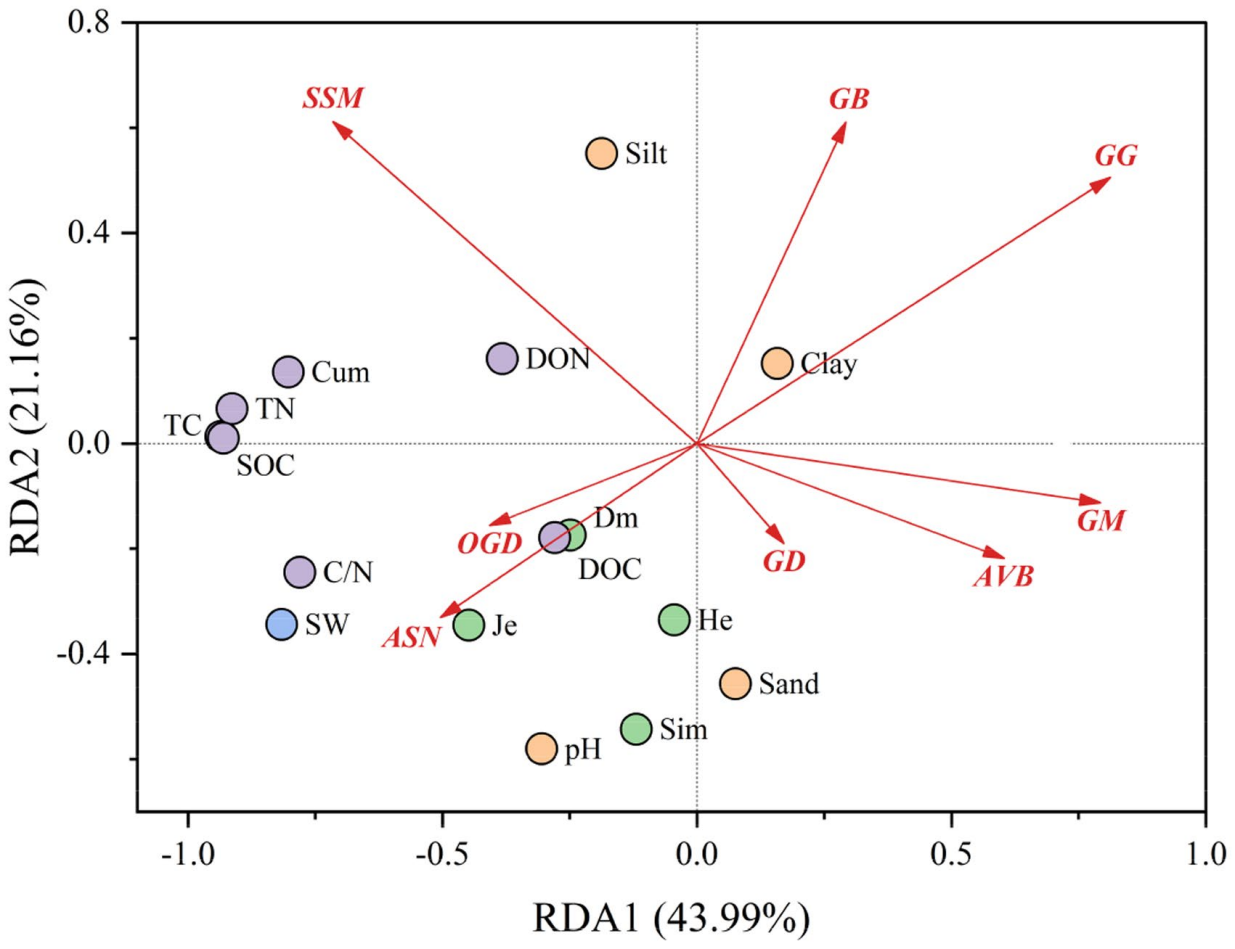
Effect of microbial SOM-degrading enzyme gene pathways on soil carbon fractions under changing water conditions. Abbreviations of major enzyme gene pathways Starch and sucrose metabolism (SSM), glycosaminoglycan degradation (GD), amino sugar and nucleotide sugar metabolism (ASN), other glycan degradation (OGD), galactose metabolism (GM), acarbose and validamycin biosynthesis (AVB), n-glycan biosynthesis (NGB), glycolysis/Gluconeogenesis (GG).

We targeted the degradative enzymes significantly associated with soil carbon fractions to discover their enzyme gene pathways and investigated the effects of microbial SOM-degrading enzyme gene pathways on soil carbon fractions under changing water conditions using RDA (Figure 7). A total of 43.99% of the variance was explained by RDA1, 21.16% by RDA2, and 65.15% by the two-axis cumulative variance. The relationships between the major enzyme gene pathways of different SOM degradation and the various influencing factors are shown in Supplementary Figure S2. The results showed that the correlations of the three pathways, OGD, ASN, and GG, with plant factors (Dm, Je), soil carbon and nitrogen fractions (DOC, C/N), and soil water were all present; whereas OGD and ASN exhibited positive correlations, and GG exhibited a negative correlation. In addition, ASN also had a negative effect on the soil physicochemical property factor Clay, and GG had a positive effect on Clay. The relationships between SSM and AVB and DON showed positive and negative correlations, respectively.

## 4. Discussion

### 4.1. Characterization of soil carbon fractions in peatlands in response to water changes

Soil organic carbon (SOC) is one of the most significant indicators of soil quality, and the interaction between SOC’s component composition and the environment is of great importance in determining the stability of soil carbon. SOC was significantly and positively correlated with changes in soil water conditions (*p* < 0.05, Figure 2); DOC had a maximum value under water-scarce wetland conditions, indicating that soil carbon fractions in peatlands responded sensitively to changes in soil water conditions. This is consistent with previous findings that as soil water in peatlands decreases, on the one hand, it reduces plant productivity, leading to a decrease in apomictic input decomposition (Deng et al., 2021). In contrast, the increase in O_2_ utilization rate and microbial respiratory activity, which contributed to the decomposition of cellulose into polysaccharides, increased the rate of enzyme reaction, which increased enzyme activity and the microbial consumption of carbon and nitrogen from plant litter. In addition to accelerating the oxidative decomposition of soil SOC, the stimulation of microbial activity decreased peatland carbon storage (Strack et al., 2015; Parvin et al., 2018).

In addition, studies have demonstrated that moderate wet-dry alternation increases the soil microbial population and promotes soil organic carbon mineralization (Huang, 2012; Zhang S. et al., 2023), thereby accelerating the decomposition of soil active carbon pools (Wang et al., 2013; Meng et al., 2015), like the findings of this study. Changes in water conditions have significant effects on SOC and DOC stored in peatlands, and these effects are indirectly related to microorganisms; however, the exact relationship is unknown and needs to be confirmed through more targeted experiments.

### 4.2. SOM degradation-associated CAZyme family and functional genes are sensitive to changes in water gradients

Organic matter in peatlands is primarily formed by depositing large amounts of plant residues, GHs and AAs are crucial to decomposing plant biomass in soil organic matter. Cellulose, hemicellulose, and lignin form a complex and stable matrix in plant biomass (Kallenbach et al., 2016). Glycoside hydrolases are primarily involved in the breakdown of polysaccharides. It hydrolyzes glycosidic bonds between two or more carbohydrate molecules or between carbohydrate molecules and non-carbohydrate fractions. AAs are oxidoreductases that work with CAZymes to degrade lignin primarily in plant cell walls (Drula et al., 2022). To further clarify the effect of water conditions on soil microorganisms. The differences in the abundance changes of the major phyla in GH and AA under different water conditions were analyzed further to elucidate the effect of water conditions on soil microorganisms. The results revealed that the changing water gradient significantly affected the abundance of three phyla: Chloroflexi, Gemmatimonadetes, and Verrucomicrobia. All of them were significantly increased under conditions of a dried wetland conditions, and the abundance of microorganisms was relatively low, as opposed to increasing with water. Chloroflexi differed significantly in both AA and GH in particular.

Gemmatimonadetes and Verrucomicrobia are reported to be the most dominant bacterial phyla in the dryland soils of goatgrass and big needlegrass in the no-water flow zone (Fawaz, 2013). It provides scientific support for the dynamic characterization that Gemmatimonadetes and Verrucomicrobia both attained their highest abundance under conditions of a dried wetland. Wang et al. (2022) found that Chloroflexi was the most dominant bacterial phylum in the seasonal water flow area, which was contrary to our findings. The reason may be related to the fact that Chloroflexus also has a photo-autotrophic nutritional mode, whereas under aerobic conditions, it exhibits chemo-heterotrophic characteristics, and under eutrophic conditions, it can grow photo-heterotrophically on simple organic matter (Shih et al., 2017). In light of this, we emphasize that the effect of water on soil bacteria is not linear and that reduced water inhibits the survival of anaerobic microorganisms. However, these microorganisms can grow better in the presence of organic matter through photo-energetic heterotrophy, and under aerobic conditions, they can use oxygen for rapid growth through the tricarboxylic acid cycle efficiently in energy production, thereby increasing microbial abundance (Martin et al., 2007). Yang et al. (2021) conducted a precipitation test in sheep grass (*Leymus chinensis*) meadow grassland. They found increased precipitation can rapidly wet the soil, and increased soil water can decrease bacterial diversity when osmotic stress is present. Although the annual precipitation and soil background water content in their study area were lower than those in the current test area, the study results were comparable in that an increase in water can significantly reduce the soil bacterial abundance in the soil. In summary, water conditions can significantly impact the microbial phylum associated with carbon catabolic enzymes in peatlands. Nonetheless, the functional gene differences of CAZyme for specific SOM degradation have yet to be entirely understood. To understand the differences between CAZyme functional genes for SOM degradation more deeply, we further investigated the changes in different SOM degradation-related CAZyme functional genes under the influence of various water conditions.

Soil enzymes are synthesized and released by microorganisms (bacteria and fungi) and plant roots (Wang et al., 2023). In the plateau peatlands, for the decomposition of different SOM fractions, the effects of microbial CAZyme genes by changes in water conditions mainly focused on plant-derived biomass and fungal-derived biomass degradation, whereas CAZyme functional genes’ effects on decomposing bacterial-derived biomass were insignificant (Figures 4, 5). Under dried wetland conditions, the abundance of CAZyme functional genes involved in plant-derived hemicellulose degradation and fungal-derived glucan degradation increased significantly, indicating that the relevant CAZymes involved in plant and fungal biomass degradation are sensitive to these conditions.

Similar results have been found in numerous other studies regarding the response of soil degradative enzymes to changes in soil water conditions. For example, in moist organic soils, drought conditions increased the activity of hydrolytic enzymes, and the rate of stabilized carbon cycling was higher in dry soils than in moist soils (Kardol et al., 2010; Kwon et al., 2013). Yan et al. (2020) simulated an extreme drought event on the Zoje Plateau and found that drought converted the typically anaerobic peat surface to aerobic, significantly increased soil hydrolase activity, and promoted an increase in the rate of soil organic carbon decomposition in the peatland (Kwon et al., 2013). The abundance of functional genes of CAZymes involved in the degradation of fungal-derived chitin was significantly lower under dried wetland conditions than under water-rich wetland conditions, which may be attributed to the changes in soil microorganisms caused by the reduction of water, thereby influencing the soil physicochemical factors of the peatland, that the dried wetland conditions altered the anaerobic environment of the soil, and increased microbial consumption of stored carbon and nitrogen in the peatland by enhancing of soil enzyme activities and increasing microbial consumption of stored carbon and nitrogen in the peatland, which in turn reduced SOC and TN content (Figure 2, *p* < 0.05). Meanwhile, in the biomass degradation of fungal-derived chitin, the degradation contribution of community GH109 (α-N-acetylglucosaminidase) was dominant (Figure 4D and Supplementary Table S1). Related studies have shown a significantly positive relationship between acetylglucosaminidase activity and soil organic carbon and nitrogen content (Ekenler and Tabatabai, 2002; Ekenler and Tabatabai, 2003). Thus, it is evident that changes in water conditions indirectly influenced the chitinase activity by influencing changes in soil carbon and nitrogen content, resulting in substantial changes in the abundance of functional genes of CAZymes involved in the degradation of fungal-derived chitin.

In addition to differences in the functional genes of CAZymes involved in plant and fungal degradation, one of the significant relationships between soil carbon fractions and plant-derived degradation-associated enzymes containing functional genes of CAZymes was observed. It may be due to the large amount of plant residue found in peat wetlands along the eastern margin of the Tibetan Plateau, which is the soil’s primary source of organic matter. Plant tissues are the most likely to contain a relatively high proportion of carbon pools (Fry et al., 2018). Soil microorganisms degrade plant biomass to form organic matter so that soil organic carbon is permanently and stably stored in soil particles and soil organic matter, reducing atmospheric CO_2_ and playing an essential role in the negative feedback regulation of the global carbon cycle (Bai and Cotrufo, 2022).

Furthermore, a significant negative correlation was primarily observed between plant-derived degradative enzymes and SOC. It may result from the degradation of peat wetlands at the eastern edge of the Tibetan Plateau due to climate change and, in some areas, decreased water levels. The decrease of vegetation cover on the peatland surface, the change of vegetation community composition and structure, the gradual decrease of biomass, and the entry of soil enzymes from the anoxic environment of water-rich wetland to the reoxygenated environment led to an increase in enzyme activities, accelerated SOC mineralization, and decreased accumulation (Wenchang et al., 2017; Wu et al., 2018). It suggests that an adequate water environment protects the organic carbon stored in peatland soils more effectively.

### 4.3. Major enzymes and metabolic pathways involved in SOM degradation

Previous research has demonstrated that changes in water conditions significantly impact soil carbon fractions (DOC and SOC). In addition, the functional genes of various SOM-degrading CAZymes were proportionally sensitive to changes in water conditions, with plant-derived degrading enzymes being significantly related to soil carbon fractions, indicating that changes in water conditions have a significant impact on the process of microbial degradation of soil organic matter in peatlands.

Further study of the carbon degrading enzyme gene pathways significantly associated with soil carbon fractions revealed that glycolysis/gluconeogenesis (GG), other glycan degradation (OGD), and amino sugar and nucleotide sugar metabolism (ASN), indirectly affect changes in soil carbon fractions by being affected by changes in water conditions and are significantly correlated with soil water, C/N, and soluble organic carbon (DOC). Importantly, glycolysis/gluconeogenesis (GG), other glycan degradation (OGD), and amino sugar and nucleotide sugar metabolism (ASN) were associated with plant- derived functional genes containing CAZyme degradative enzymes, suggesting a stronger indirect effect of carbohydrate- active enzymes related with the degradation of plant- derived biomass on soil carbon fractions. It has also been demonstrated that plant- derived degradative enzymes have a stronger effect on organic matter degradation in water-dry soils, and that the enzyme activity is significantly affected by changes in water content, which indirectly enhances the degradation of cellulosic compounds by increasing the oxygen content to open the ‘enzyme lock’ (Moorcroft et al., 2008; Guangyuan et al., 2020), which is similar to our results.

Wiedermann et al. (2017) suggested that soil hydrolases decreased under drought conditions, which might be due to the different composition of plant communities in the study site. Wiedermann et al. (2017) concluded that the plant communities in the sample sites were dominated by Rhododendron and other mycorrhizal woody shrubs and trees, and that at low water, Rhododendron mycorrhizae might reduce the action of hydrolases by limiting the positive effects of increased oxygen on decomposition. In contrast, our sampling sites were all sedge grasslands, which are plants mainly in the gramineae and sedge family as the establishment species, in which cellulose, hemicellulose, and other plant biomass content is higher (Zhang et al., 2012), and the degradation of plant-derived degradative enzymes is stronger under drought conditions, which in turn increases the degradation of soil organic carbon (Moorcroft et al., 2008). This shows that water conditions can regulate soil organic carbon degradation processes by affecting carbohydrate- active enzymes related to the degradation of plant-derived biomass, and further emphasizes the importance of plant-derived biomass decomposition in regulating carbon cycling in plateau peatland ecosystems.

## 5. Conclusion

This study confirmed that soil carbon fractions and microbial CAZyme functional genes are sensitive to changes in water conditions in plateau wetland ecosystems. We discovered that the microbial selection of soil carbon sources differs under various water conditions. Specifically, microorganisms can respond to water changes by selectively regulating CAZyme functional genes in plant and fungal carbon sources. Further analysis determined that the microbial treatment of plant-derived carbon in plateau peat wetlands significantly influenced carbon pools in terms of individual soil enzymes and carbon metabolic pathways. However, there are still some limitations to be addressed in this study: (1) plant biomass degradation was investigated in this study, but it was not linked to the aboveground and belowground biomass of wetland plants; and (2) drought was suggested to increase the release of soil organic carbon from peatlands in this study, but more in-depth studies are required across a broader range of temperature gradients and time scales.

## Data availability statement

The datasets presented in this study can be found in online repositories. The names of the repository/repositories and accession number(s) can be found at: https://www.ncbi.nlm.nih.gov/, PRJNA997629.

## Author contributions

MX: Data curation, Writing – original draft. WJ: Data curation, Investigation, Writing – review & editing. SZ: Investigation, Writing – review & editing, Software. DK: Investigation, Software, Writing – review & editing, Data curation, Funding acquisition, Writing – original draft. XY: Writing – review & editing.

## Funding

The author(s) declare financial support was received for the research, authorship, and/or publication of this article. The author(s) declare financial support was received for the Natural Science Foundation of Sichuan Province (2023NSFSC0143) and the National Natural Science Foundation of China (31800458) of this article.

## Conflict of interest

The authors declare that the research was conducted in the absence of any commercial or financial relationships that could be construed as a potential conflict of interest.

## Publisher’s note

All claims expressed in this article are solely those of the authors and do not necessarily represent those of their affiliated organizations, or those of the publisher, the editors and the reviewers. Any product that may be evaluated in this article, or claim that may be made by its manufacturer, is not guaranteed or endorsed by the publisher.
