# Supplementary material for: Microbial carbohydrate-active enzymes influence soil carbon by regulating the of plant- and fungal-derived biomass decomposition in plateau peat wetlands under differing water conditions
Source: Front Microbiol. 2023 Sep 4;14:1266016. doi: 10.3389/fmicb.2023.1266016 (PMC10507343; doi:10.3389/fmicb.2023.1266016)
Supplement: Supplementary file 1 [file Data_Sheet_1.pdf]

Supplementary Material

Part S1. Measurement of cumulative soil respiration (C_{um})

A soil sample and a small beaker containing 15 ml of 1 mol/L NaOH were placed in a sealed bottle and placed in a climatic incubator for sealed incubation; measurements were made on the basis of the number of days of the experiment (0, 1, 2, 4, 7, 11, 15, 20, and 28 days), and 60% water content was adjusted at each measurement. This was done under two groups of incubation conditions A and B, respectively.

A: 14.5°C, 13h during the day; 8°C, 11h at night (low temperature)

B: 19.5°C, 13h during the day; 13°C, 11h at night (high temperature)

Soil cumulative respiration (C_{um}) is first phenolphthalein as an indicator, HCl titration to neutralize the remaining NaOH, so that the generation of Na_2CO_3 transformed into $NaHCO_3$, and then methyl orange as an indicator, HCl titration, so that all the $NaHCO_3$ are changed into NaCl, methyl orange as an indicator when the amount of HCl consumed by two times, that is, the neutralization of the Na_2CO_3 dosage, so that the absorption of the CO_2 amount can be calculated. Finally the C_{um} value by the formula.

(1) Formula for weight of CO_2 in solution is as follows (W, g):

$$W = (V1 - V2) \times C \times \frac{44}{2\times1000} \times \frac{250}{25} \quad (1)$$

where V1 is two times the number of milliliters (ml) of HCI used for the test solution with methyl orange as indicator; V2 is two times the number of milliliters (ml) of HC1 used for the blank test solution with methyl orange as indicator; C is the molar concentration of HCl (mol/L); is the millimolar mass of CO_2; and is the fractional multiplier.

Table S1. Functional classification of glycosyl hydrolases (GH) and auxilliary (AA) encoding the enzymatic activities involved in the plant-and microbial com pounds degradation according to CAZy.

Group	Compound	CAZy families (GH and AA)
Plant biomass	Cellulose	GH13(α-amylase),GH105(α-amylase),GH57(α-amylase), GH88(α-amylase),GH63(α-glucosidase),GH15(glucoamy lase),GH4(α-glucosidase),GH97(α-glucosidase),GH33(gl ucan phosphorylase),GH77(amylomaltase),GH1 (β-glucosidase),GH3 (βglucosidase),GH5 (β-glucosidase/endoglucanase),GH8 (endoglucanase/ endoxylanase),GH9 (endoglucanase),GH116 (β-glucosidase),AA10 (lytic polysaccharide monooxygenase),GH94(cellobiose phosphorylase),GH42(β-galactosidase),GH92 (1,2-α-mannosidase)

	Hemicellulose	GH2 (β-galactosidase/β-glucuronidase),GH10 (endoxylanase),GH11 (endoxylanase),GH30 (endoxylanase/β-1,6- glucanase/β-xylosidase),GH36 (α-galactosidase),GH39 (β-xylosidase/α-L-arabinofuranosidase),GH43 (β-xylosidase/endoxylanase),GH51 (α-L_x0002_arabinofuranosidase),GH54 (α-L-arabinofuranosidase), GH74 (xyloglucanase),GH95 (α-L-fucosidase/α-L-galactosidase),GH115 (xylan α-1,2-glucuronidase),GH38(α-mannosidase),GH125(1,6-α-D-mannosidase),GH99(endo-α-D-mannosidase),GH67(α-glucuronidase),GH47(α-mannosidase),GH28(polygalacturonase),GH59(β-galactosidase),GH53(endo-β-1,4-galactanase),GH35(β-galactosidase),
	Lignin	AA2 (peroxidase),AA3 (oxidase),AA4 (oxidase),AA6 (1,4- benzoquinone reductase),AA7(glucooligosaccharide oxidase)
Fungal biomass	Chitin	GH16 (xyloglucanase/endoglucanase),GH20 (N-acetylβ-glucosaminidase),GH89(α-N-acetylglucosaminidase),GH109(α-N-acetylglucosaminidase),GH108 (lysozyme)
	Glucans	GH17 (endo-1,3-β-glucanase),GH31(α-glucosidase)
Bacterial biomass	Peptidoglycan	GH23 (lysozyme/ peptidoglycan lytic transglycosylase),GH24 (lysozyme),GH73 (peptidoglycan hydrolase with endo-β-Nacetylglucosaminidase specificity),GH102 (peptidoglycan lytic transglycosylase),GH103 (peptidoglycan lytic transglycosylase),
Others	others	GH37,GH78,GH106,GH65,GH29

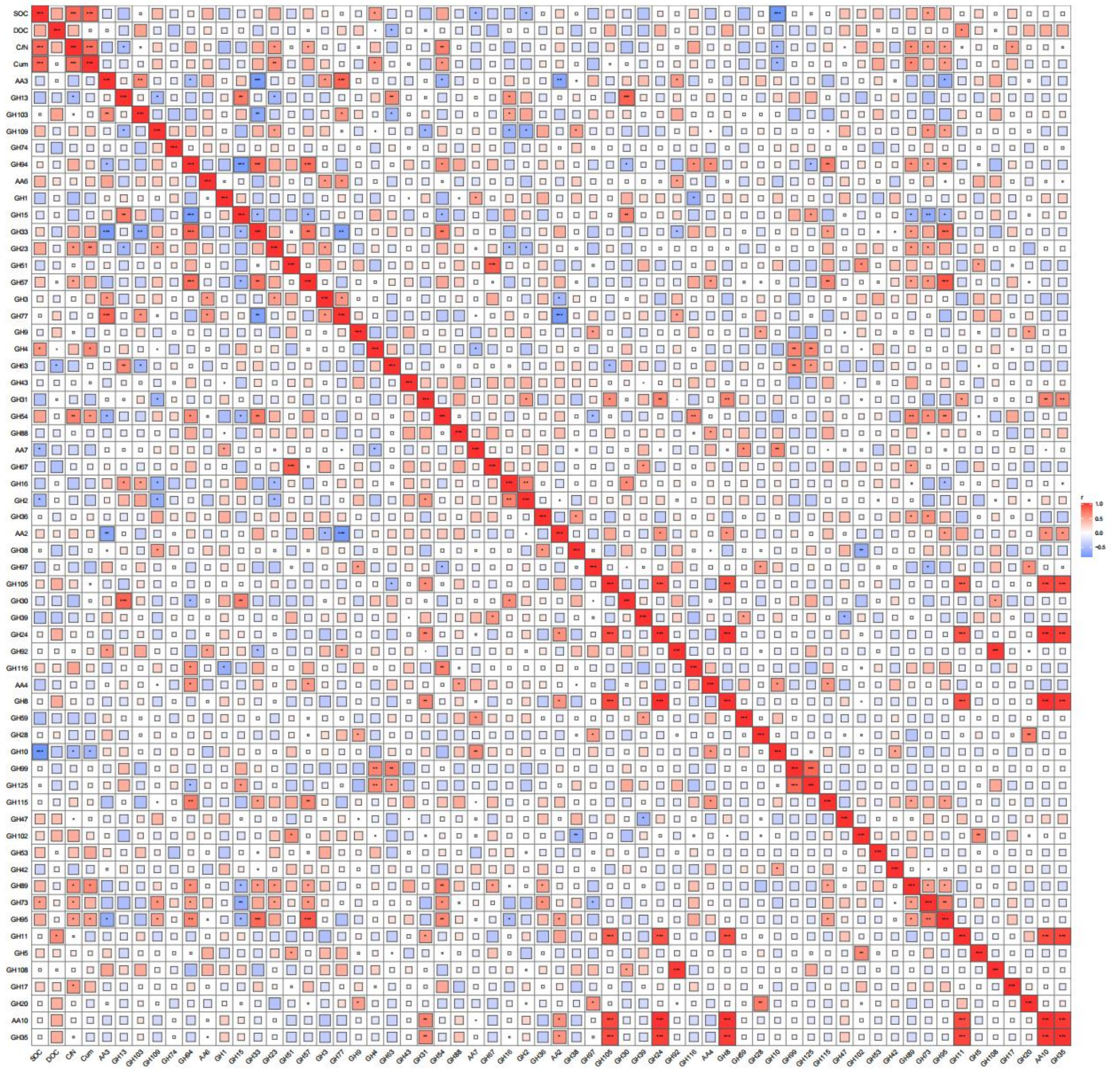

Fig. S1. Correlation between soil carbon components and CAZymes functional genes of carbon decomposition .

Table S2.The carbon metabolism pathways of the related soil enzyme which mainly involved in carbon degradation

Compound	enzyme	metabolic pathway
Cellulose	maltose-6-phosphate glucosidase (EC 3.2.1.122)	Starch and sucrose metabolism
Glucans	Glucan endo-1,3-beta-glucosidase (EC 3.2.1.39)	
Peptidoglycan	peptidoglycan lytic transglycosylase (EC 3.2.1.-)	Glycosaminoglycan degradation
Chitin	alpha-N-acetylglucosaminidase (EC 3.2.1.50)	
Hemicellulose	alpha-L-arabinofuranosidase(EC 3.2.1.55)	Amino sugar and nucleotide sugar metabolism
Peptidoglycan	lysozyme (EC 3.2.1.17)	
Hemicellulose	alpha-L-fucosidase(EC 3.2.1.51)	Other glycan degradation
Hemicellulose	beta-galactosidase(EC 3.2.1.23)	Galactose metabolism
Lignin	glucooligosaccharide oxidase(EC 1.1.3.-)	Acarbose and validamycin biosynthesis
Cellulose	processing alpha-glucosidase (EC 3.2.1.106)	N-Glycan biosynthesis
Hemicellulose	endo-beta-1,4-xylanase (EC 3.2.1.8)	Glycolysis /Gluconeogenesis

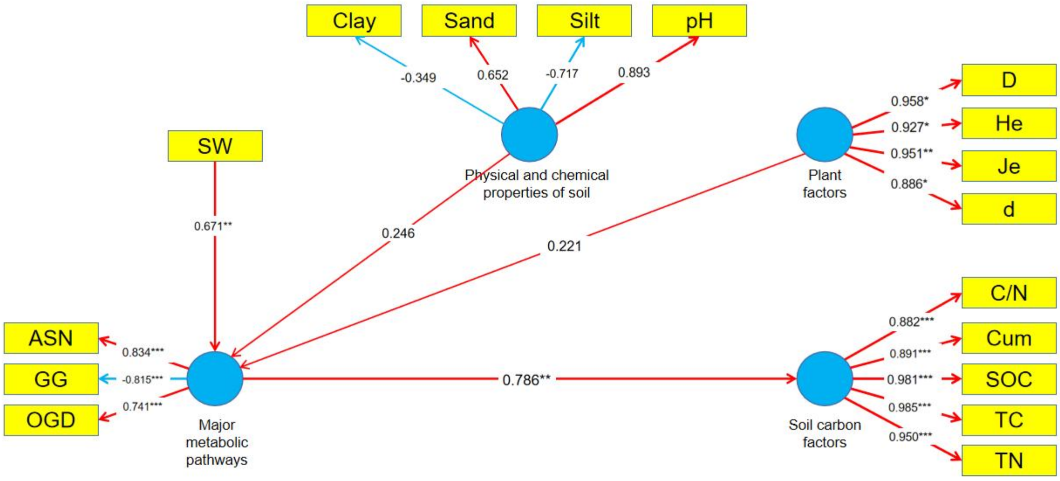

Fig. S2.Major structural equation models for different SOM degradation
